# {*N*,*N*′-Bis[1-(2-pyrid­yl)ethyl­idene]propane-1,2-diamine-κ^4^ *N*,*N*′,*N*′′,*N*′′′}bis­(thio­cyanato-κ*N*)manganese(II)

**DOI:** 10.1107/S1600536810021549

**Published:** 2010-06-16

**Authors:** Fu-Ming Wang

**Affiliations:** a Department of Chemistry, Dezhou University, Dezhou Shandong 253023, People’s Republic of China

## Abstract

In the title compound, [Mn(NCS)_2_(C_17_H_20_N_4_)], the Mn^II^ atom is six-coordinated by the *N*,*N*′,*N*′′,*N*′′′-tetra­dentate Schiff base ligand and by two *trans*-N atoms from two thio­cyanate anions, forming a distorted octa­hedral geometry. The dihedral angle between the aromatic rings of the Schiff base is 9.5 (3)°.

## Related literature

For another complex containing 1,2-bis­(2′-pyridyl­methyl­ene­amino)­propane, see: Ouyang *et al.* (2002 ▶). For related manganese(II) complexes with Schiff bases, see: Louloudi *et al.* (1999 ▶); Sra *et al.* (2000 ▶); Karmakar *et al.* (2005 ▶); Deoghoria *et al.* (2005 ▶). For the synthesis of the Schiff base, see: Gourbatsis *et al.* (1990 ▶).
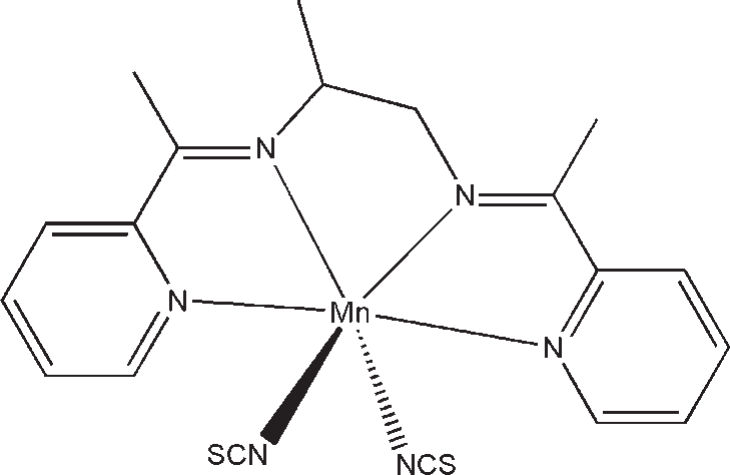


## Experimental

### 

#### Crystal data

[Mn(NCS)_2_(C_17_H_20_N_4_)]
*M* *_r_* = 451.47
Triclinic, 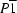

*a* = 8.647 (3) Å
*b* = 9.135 (2) Å
*c* = 14.608 (3) Å
α = 84.701 (3)°
β = 79.407 (3)°
γ = 70.509 (3)°
*V* = 1068.6 (5) Å^3^
*Z* = 2
Mo *K*α radiation
μ = 0.83 mm^−1^
*T* = 298 K
0.33 × 0.30 × 0.30 mm

#### Data collection

Bruker SMART CCD diffractometer
Absorption correction: multi-scan (*SADABS*; Sheldrick, 1996 ▶) *T*_min_ = 0.771, *T*_max_ = 0.789
11100 measured reflections
4608 independent reflections
2211 reflections with *I* > 2σ(*I*)
*R*_int_ = 0.078

#### Refinement

*R*[*F* ^2^ > 2σ(*F* ^2^)] = 0.077
*wR*(*F* ^2^) = 0.224
*S* = 0.99
4608 reflections
256 parameters
H-atom parameters constrained
Δρ_max_ = 0.68 e Å^−3^
Δρ_min_ = −0.34 e Å^−3^

### 

Data collection: *SMART* (Bruker, 1998 ▶); cell refinement: *SAINT* (Bruker, 1998 ▶); data reduction: *SAINT*; program(s) used to solve structure: *SHELXS97* (Sheldrick, 2008 ▶); program(s) used to refine structure: *SHELXL97* (Sheldrick, 2008 ▶); molecular graphics: *SHELXTL* (Sheldrick, 2008 ▶); software used to prepare material for publication: *SHELXTL*.

## Supplementary Material

Crystal structure: contains datablocks global, I. DOI: 10.1107/S1600536810021549/hb5486sup1.cif

Structure factors: contains datablocks I. DOI: 10.1107/S1600536810021549/hb5486Isup2.hkl

Additional supplementary materials:  crystallographic information; 3D view; checkCIF report

## Figures and Tables

**Table 1:** Selected bond lengths (Å)

Mn1—N5	2.127 (6)
Mn1—N6	2.149 (6)
Mn1—N2	2.258 (5)
Mn1—N3	2.260 (4)
Mn1—N4	2.334 (4)
Mn1—N1	2.346 (4)

**Fig. 1.**
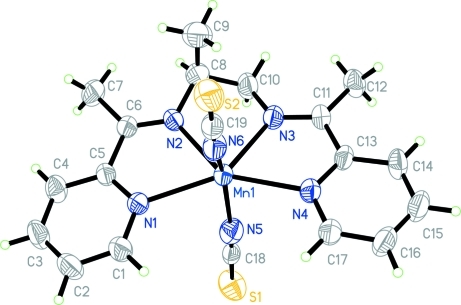
The molecular structure of (I), showing displacement ellipsoids drawn at the 30% probability level.

## supplementary crystallographic information

### Comment

Metal complexes with Schiff bases have been known since 1840 but only one complex derived from 1,2-bis(2'-pyridylmethyleneamino)propane has been reported (Ouyang *et al.*, 2002). In this paper, the title new manganese(II) complex is reported.

In the title complex, Fig. 1, the Mn^II^ atom is six-coordinated by four N atoms of the Schiff base ligand 1,2-bis(2'-pyridylmethyleneamino)propane, and by two N atoms from two thiocyanate ligands, forming a distorted octahedral geometry. The coordinate bond lengths (Table 1) are comparable with those observed in other similar manganese(II) complexes with Schiff bases (Louloudi *et al.*, 1999; Sra *et al.*, 2000; Karmakar *et al.*, 2005; Deoghoria *et al.*, 2005).

### Experimental

The Schiff base ligand 1,2-bis(2'-pyridylmethyleneamino)propane was synthesized according to the literature method (Gourbatsis *et al.*, 1990). To a stirred methanol solution of the Schiff base ligand (1.0 mmol, 0.280 g) was added a methanol solution of manganese acetate (1.0 mmol, 0.245 g) and ammonium thiocyanate (1.0 mmol, 0.076 g). The mixture was boiled under reflux for 2 h, then cooled to room temperature. Brown blocks of (I) were formed after slow evaporation of the solution in air for a few days.

### Refinement

Hydrogen atoms were placed in geometrically idealized positions and constrained to ride on their parent atoms, with C–H distances of 0.93–0.97 Å, and with *U*_iso_(H) set at 1.2*U*_eq_(C) and 1.5*U*_eq_(C_methyl_).

### Crystal data

**Table:**

[Mn(NCS)_2_(C_17_H_20_N_4_)]	*Z* = 2
*M**_r_* = 451.47	*F*(000) = 466
Triclinic, *P*1	*D*_x_ = 1.403 Mg m^−^^3^
Hall symbol: -P 1	Mo *K*α radiation, λ = 0.71073 Å
*a* = 8.647 (3) Å	Cell parameters from 1307 reflections
*b* = 9.135 (2) Å	θ = 2.3–24.5°
*c* = 14.608 (3) Å	µ = 0.83 mm^−^^1^
α = 84.701 (3)°	*T* = 298 K
β = 79.407 (3)°	Block, brown
γ = 70.509 (3)°	0.33 × 0.30 × 0.30 mm
*V* = 1068.6 (5) Å^3^	

### Data collection

**Table:**

Bruker SMART CCD diffractometer	4608 independent reflections
Radiation source: fine-focus sealed tube	2211 reflections with *I* > 2σ(*I*)
graphite	*R*_int_ = 0.078
ω scan	θ_max_ = 27.0°, θ_min_ = 2.4°
Absorption correction: multi-scan (*SADABS*; Sheldrick, 1996)	*h* = −11→11
*T*_min_ = 0.771, *T*_max_ = 0.789	*k* = −11→11
11100 measured reflections	*l* = −18→18

### Refinement

**Table:**

Refinement on *F*^2^	Primary atom site location: structure-invariant direct methods
Least-squares matrix: full	Secondary atom site location: difference Fourier map
*R*[*F*^2^ > 2σ(*F*^2^)] = 0.077	Hydrogen site location: inferred from neighbouring sites
*wR*(*F*^2^) = 0.224	H-atom parameters constrained
*S* = 0.99	*w* = 1/[σ^2^(*F*_o_^2^) + (0.1044*P*)^2^] where *P* = (*F*_o_^2^ + 2*F*_c_^2^)/3
4608 reflections	(Δ/σ)_max_ < 0.001
256 parameters	Δρ_max_ = 0.68 e Å^−^^3^
0 restraints	Δρ_min_ = −0.33 e Å^−^^3^

### Special details

**Table:**

Geometry. All e.s.d.'s (except the e.s.d. in the dihedral angle between two l.s. planes) are estimated using the full covariance matrix. The cell e.s.d.'s are taken into account individually in the estimation of e.s.d.'s in distances, angles and torsion angles; correlations between e.s.d.'s in cell parameters are only used when they are defined by crystal symmetry. An approximate (isotropic) treatment of cell e.s.d.'s is used for estimating e.s.d.'s involving l.s. planes.
Refinement. Refinement of *F*^2^ against ALL reflections. The weighted *R*-factor *wR* and goodness of fit *S* are based on *F*^2^, conventional *R*-factors *R* are based on *F*, with *F* set to zero for negative *F*^2^. The threshold expression of *F*^2^ > σ(*F*^2^) is used only for calculating *R*-factors(gt) *etc*. and is not relevant to the choice of reflections for refinement. *R*-factors based on *F*^2^ are statistically about twice as large as those based on *F*, and *R*- factors based on ALL data will be even larger.

### Fractional atomic coordinates and isotropic or equivalent isotropic displacement parameters (Å^2^)

**Table:**

	*x*	*y*	*z*	*U*_iso_*/*U*_eq_	
Mn1	1.02210 (10)	0.54382 (9)	0.27241 (5)	0.0601 (3)	
N1	1.2061 (6)	0.6565 (5)	0.3134 (3)	0.0644 (12)	
N2	1.0220 (6)	0.7580 (5)	0.1819 (3)	0.0673 (12)	
N3	0.8157 (6)	0.5912 (5)	0.1875 (3)	0.0701 (13)	
N4	0.9013 (5)	0.3471 (5)	0.3008 (3)	0.0665 (12)	
N5	0.8737 (7)	0.6394 (7)	0.3994 (4)	0.0906 (17)	
N6	1.2282 (6)	0.3744 (6)	0.1940 (4)	0.0772 (15)	
S1	0.6757 (2)	0.8093 (2)	0.54416 (16)	0.1097 (7)	
S2	1.4735 (2)	0.1575 (2)	0.08162 (12)	0.0855 (5)	
C1	1.2937 (8)	0.6060 (8)	0.3826 (5)	0.089 (2)	
H1	1.2883	0.5148	0.4151	0.107*	
C2	1.3917 (9)	0.6811 (8)	0.4088 (5)	0.096 (2)	
H2	1.4508	0.6422	0.4578	0.116*	
C3	1.3997 (8)	0.8144 (8)	0.3608 (5)	0.088 (2)	
H3	1.4654	0.8677	0.3765	0.106*	
C4	1.3109 (7)	0.8693 (6)	0.2897 (5)	0.0750 (17)	
H4	1.3155	0.9601	0.2565	0.090*	
C5	1.2135 (6)	0.7880 (6)	0.2674 (4)	0.0574 (13)	
C6	1.1124 (7)	0.8394 (7)	0.1905 (4)	0.0631 (14)	
C7	1.1270 (9)	0.9785 (7)	0.1307 (5)	0.095 (2)	
H7A	1.2372	0.9548	0.0956	0.142*	
H7B	1.1054	1.0646	0.1696	0.142*	
H7C	1.0477	1.0050	0.0888	0.142*	
C8	0.9234 (8)	0.7889 (8)	0.1062 (4)	0.087 (2)	
H8	0.8913	0.8996	0.0880	0.104*	
C9	1.0322 (10)	0.6917 (9)	0.0230 (5)	0.106 (2)	
H9A	1.1312	0.7198	0.0042	0.159*	
H9B	0.9712	0.7114	−0.0281	0.159*	
H9C	1.0619	0.5833	0.0409	0.159*	
C10	0.7743 (8)	0.7434 (8)	0.1372 (5)	0.098 (2)	
H10A	0.7250	0.7379	0.0837	0.118*	
H10B	0.6935	0.8213	0.1779	0.118*	
C11	0.7315 (7)	0.4988 (6)	0.1900 (4)	0.0617 (14)	
C12	0.5892 (7)	0.5241 (7)	0.1393 (5)	0.0843 (19)	
H12A	0.5809	0.6123	0.0970	0.126*	
H12B	0.4880	0.5428	0.1833	0.126*	
H12C	0.6072	0.4335	0.1050	0.126*	
C13	0.7763 (6)	0.3606 (6)	0.2541 (4)	0.0613 (14)	
C14	0.6956 (7)	0.2485 (8)	0.2671 (5)	0.0826 (18)	
H14	0.6105	0.2573	0.2340	0.099*	
C15	0.7432 (8)	0.1242 (8)	0.3297 (5)	0.094 (2)	
H15	0.6899	0.0495	0.3389	0.112*	
C16	0.8672 (9)	0.1124 (8)	0.3771 (5)	0.094 (2)	
H16	0.9006	0.0303	0.4195	0.112*	
C17	0.9429 (8)	0.2251 (7)	0.3610 (4)	0.0808 (18)	
H17	1.0283	0.2165	0.3937	0.097*	
C18	0.7938 (7)	0.7096 (6)	0.4577 (4)	0.0589 (14)	
C19	1.3320 (7)	0.2828 (7)	0.1471 (4)	0.0631 (15)	

### Atomic displacement parameters (Å^2^)

**Table:**

	*U*^11^	*U*^22^	*U*^33^	*U*^12^	*U*^13^	*U*^23^
Mn1	0.0670 (6)	0.0649 (6)	0.0607 (5)	−0.0367 (5)	−0.0173 (4)	0.0090 (4)
N1	0.082 (3)	0.060 (3)	0.064 (3)	−0.036 (2)	−0.024 (2)	0.011 (2)
N2	0.078 (3)	0.075 (3)	0.066 (3)	−0.041 (3)	−0.030 (2)	0.012 (2)
N3	0.075 (3)	0.072 (3)	0.077 (3)	−0.039 (3)	−0.026 (3)	0.017 (3)
N4	0.063 (3)	0.077 (3)	0.071 (3)	−0.036 (2)	−0.018 (2)	0.009 (3)
N5	0.089 (4)	0.110 (5)	0.082 (4)	−0.051 (4)	−0.001 (3)	−0.006 (3)
N6	0.079 (4)	0.077 (4)	0.086 (4)	−0.040 (3)	−0.016 (3)	0.003 (3)
S1	0.0955 (14)	0.1071 (16)	0.1234 (16)	−0.0214 (12)	−0.0135 (12)	−0.0410 (13)
S2	0.0850 (12)	0.0822 (12)	0.0928 (12)	−0.0285 (10)	−0.0153 (9)	−0.0139 (9)
C1	0.116 (5)	0.088 (5)	0.091 (5)	−0.060 (4)	−0.049 (4)	0.026 (4)
C2	0.114 (5)	0.099 (5)	0.102 (5)	−0.051 (5)	−0.063 (4)	0.019 (4)
C3	0.096 (5)	0.078 (5)	0.115 (5)	−0.047 (4)	−0.047 (4)	0.001 (4)
C4	0.080 (4)	0.052 (3)	0.103 (5)	−0.029 (3)	−0.030 (4)	0.003 (3)
C5	0.055 (3)	0.060 (3)	0.062 (3)	−0.025 (3)	−0.010 (3)	−0.006 (3)
C6	0.065 (3)	0.067 (4)	0.064 (3)	−0.030 (3)	−0.015 (3)	0.008 (3)
C7	0.121 (6)	0.080 (5)	0.103 (5)	−0.061 (4)	−0.037 (4)	0.039 (4)
C8	0.113 (5)	0.080 (5)	0.085 (4)	−0.049 (4)	−0.042 (4)	0.027 (4)
C9	0.143 (7)	0.102 (6)	0.083 (5)	−0.051 (5)	−0.026 (5)	0.003 (4)
C10	0.108 (5)	0.110 (6)	0.110 (5)	−0.070 (5)	−0.060 (4)	0.042 (4)
C11	0.057 (3)	0.059 (3)	0.075 (4)	−0.026 (3)	−0.011 (3)	−0.005 (3)
C12	0.065 (4)	0.088 (5)	0.112 (5)	−0.032 (3)	−0.037 (4)	0.006 (4)
C13	0.056 (3)	0.064 (4)	0.069 (3)	−0.032 (3)	0.002 (3)	−0.004 (3)
C14	0.067 (4)	0.086 (5)	0.113 (5)	−0.046 (4)	−0.021 (4)	0.000 (4)
C15	0.094 (5)	0.090 (5)	0.113 (6)	−0.059 (4)	−0.015 (4)	0.022 (4)
C16	0.099 (5)	0.089 (5)	0.106 (5)	−0.054 (4)	−0.022 (4)	0.034 (4)
C17	0.087 (4)	0.086 (5)	0.088 (4)	−0.047 (4)	−0.034 (4)	0.022 (4)
C18	0.057 (4)	0.052 (4)	0.075 (4)	−0.026 (3)	−0.022 (3)	0.013 (3)
C19	0.063 (4)	0.061 (4)	0.075 (4)	−0.030 (3)	−0.024 (3)	0.013 (3)

### Geometric parameters (Å, °)

**Table:**

Mn1—N5	2.127 (6)	C5—C6	1.495 (7)
Mn1—N6	2.149 (6)	C6—C7	1.501 (8)
Mn1—N2	2.258 (5)	C7—H7A	0.9600
Mn1—N3	2.260 (4)	C7—H7B	0.9600
Mn1—N4	2.334 (4)	C7—H7C	0.9600
Mn1—N1	2.346 (4)	C8—C10	1.465 (8)
N1—C1	1.331 (7)	C8—C9	1.540 (9)
N1—C5	1.336 (6)	C8—H8	0.9800
N2—C6	1.274 (6)	C9—H9A	0.9600
N2—C8	1.470 (7)	C9—H9B	0.9600
N3—C11	1.281 (6)	C9—H9C	0.9600
N3—C10	1.476 (7)	C10—H10A	0.9700
N4—C17	1.345 (7)	C10—H10B	0.9700
N4—C13	1.348 (6)	C11—C13	1.486 (8)
N5—C18	1.097 (7)	C11—C12	1.493 (7)
N6—C19	1.164 (7)	C12—H12A	0.9600
S1—C18	1.608 (7)	C12—H12B	0.9600
S2—C19	1.600 (7)	C12—H12C	0.9600
C1—C2	1.376 (8)	C13—C14	1.402 (7)
C1—H1	0.9300	C14—C15	1.389 (8)
C2—C3	1.364 (9)	C14—H14	0.9300
C2—H2	0.9300	C15—C16	1.351 (8)
C3—C4	1.364 (8)	C15—H15	0.9300
C3—H3	0.9300	C16—C17	1.376 (8)
C4—C5	1.389 (7)	C16—H16	0.9300
C4—H4	0.9300	C17—H17	0.9300
			
N5—Mn1—N6	152.6 (2)	H7A—C7—H7B	109.5
N5—Mn1—N2	102.5 (2)	C6—C7—H7C	109.5
N6—Mn1—N2	99.53 (18)	H7A—C7—H7C	109.5
N5—Mn1—N3	98.1 (2)	H7B—C7—H7C	109.5
N6—Mn1—N3	103.71 (18)	C10—C8—N2	109.6 (5)
N2—Mn1—N3	73.15 (16)	C10—C8—C9	109.9 (6)
N5—Mn1—N4	86.78 (19)	N2—C8—C9	107.8 (5)
N6—Mn1—N4	85.41 (17)	C10—C8—H8	109.8
N2—Mn1—N4	142.80 (16)	N2—C8—H8	109.8
N3—Mn1—N4	69.88 (16)	C9—C8—H8	109.8
N5—Mn1—N1	82.92 (18)	C8—C9—H9A	109.5
N6—Mn1—N1	89.82 (17)	C8—C9—H9B	109.5
N2—Mn1—N1	69.50 (15)	H9A—C9—H9B	109.5
N3—Mn1—N1	141.88 (17)	C8—C9—H9C	109.5
N4—Mn1—N1	147.69 (16)	H9A—C9—H9C	109.5
C1—N1—C5	117.9 (5)	H9B—C9—H9C	109.5
C1—N1—Mn1	125.2 (4)	C8—C10—N3	110.8 (5)
C5—N1—Mn1	116.7 (3)	C8—C10—H10A	109.5
C6—N2—C8	121.8 (5)	N3—C10—H10A	109.5
C6—N2—Mn1	122.4 (4)	C8—C10—H10B	109.5
C8—N2—Mn1	115.5 (3)	N3—C10—H10B	109.5
C11—N3—C10	122.6 (5)	H10A—C10—H10B	108.1
C11—N3—Mn1	122.1 (4)	N3—C11—C13	115.4 (5)
C10—N3—Mn1	114.9 (3)	N3—C11—C12	125.6 (5)
C17—N4—C13	117.9 (5)	C13—C11—C12	118.9 (5)
C17—N4—Mn1	125.7 (4)	C11—C12—H12A	109.5
C13—N4—Mn1	116.3 (3)	C11—C12—H12B	109.5
C18—N5—Mn1	169.2 (6)	H12A—C12—H12B	109.5
C19—N6—Mn1	174.9 (5)	C11—C12—H12C	109.5
N1—C1—C2	123.6 (6)	H12A—C12—H12C	109.5
N1—C1—H1	118.2	H12B—C12—H12C	109.5
C2—C1—H1	118.2	N4—C13—C14	120.4 (5)
C3—C2—C1	118.0 (6)	N4—C13—C11	116.3 (5)
C3—C2—H2	121.0	C14—C13—C11	123.3 (5)
C1—C2—H2	121.0	C15—C14—C13	119.7 (6)
C2—C3—C4	119.7 (6)	C15—C14—H14	120.2
C2—C3—H3	120.1	C13—C14—H14	120.2
C4—C3—H3	120.1	C16—C15—C14	119.6 (6)
C3—C4—C5	119.2 (6)	C16—C15—H15	120.2
C3—C4—H4	120.4	C14—C15—H15	120.2
C5—C4—H4	120.4	C15—C16—C17	118.2 (6)
N1—C5—C4	121.6 (5)	C15—C16—H16	120.9
N1—C5—C6	115.6 (4)	C17—C16—H16	120.9
C4—C5—C6	122.8 (5)	N4—C17—C16	124.2 (6)
N2—C6—C5	115.6 (5)	N4—C17—H17	117.9
N2—C6—C7	126.2 (5)	C16—C17—H17	117.9
C5—C6—C7	118.2 (5)	N5—C18—S1	178.7 (6)
C6—C7—H7A	109.5	N6—C19—S2	179.4 (6)
C6—C7—H7B	109.5		
